# Doings of Dentists' Convention

**Published:** 1839-08

**Authors:** 


### DOINGS OF DENTISTS' CONVENTION.

It being the opinion of the Dental Association of Western New-York, (auxilliary to the Society of Surgeon Dentists of the City of New-York) that the American Journal of Dental Science is highly calculated to exalt the character and standing of the profession in the United States :—

Therefore, *resolved*, That we, as a Society, and as individuals, will use our utmost endeavours to further the worthy objects contemplated by the enterprising editors and projectors of said Journal, by procuring subscriptions, and by contributing, as may be in our power, to its pages.

Horatio N. Fenn, M D., *Rochester*,
S. Bliss, M. D., *Syracuse.*
G. A. Foster, *Utica.*
R. Griswold, *Bainbridge.*
Drs. Hayes & Harvey, *Buffalo.*
Dr. U. H. Dunning, *Buffalo.*
E. Carr, M. D., *Canandaigua.*
Dr. D. O. Crane, *Geneva.*
Dr. Alpheus Matson, *Auburn.*
A. W. Lathrop, M. D. *Auburn.*
Dr. Blakely, *Utica.*

